# Pathological Mechanisms and Therapeutic Potential of Mitochondrial Dysfunction in Heart Failure

**DOI:** 10.31083/RCM51920

**Published:** 2026-08-21

**Authors:** Shige San, Xi Yang, Liang Shen, Kun Meng

**Affiliations:** ^1^Department of Central Laboratory, Xiangyang Central Hospital, Affiliated Hospital of Hubei University of Arts and Science, 441021 Xiangyang, Hubei, China; ^2^Department of Internal Medicine, Affiliated Hospital of Xiangyang Vocational and Technical University, 441050 Xiangyang, Hubei, China; ^3^Hubei Key Laboratory of Gynecologic Oncology and Reproductive Health, Xiangyang Central Hospital, Affiliated Hospital of Hubei University of Arts and Science, 441021 Xiangyang, Hubei, China

**Keywords:** heart failure, mitochondrial dysfunction, energy metabolism, reactive oxygen species, ferroptosis

## Abstract

Heart failure (HF) is a clinical syndrome resulting from structural or functional cardiac abnormalities and represents a growing global public health challenge. Common etiologies include myocardial infarction, cardiomyopathy, and myocarditis. Mitochondrial dysfunction is now recognized as a central event in the initiation and progression of HF, making this pathogenesis a key focus of recent research. Recent studies have confirmed that HF with reduced ejection fraction (HFrEF) is driven by defects in excitation–contraction coupling, leading to mechano-energetic uncoupling. In contrast, HF with preserved ejection fraction (HFpEF) is characterized by an imbalance between cardiac workload and mitochondrial energy supply. Mitochondrial dysfunction is evident in both forms of HF, with metabolic disturbances serving as both biomarkers and drivers of disease progression. However, key knowledge gaps remain, including uncertainty regarding the regulation of mitochondrial networks and the limited clinical translation of targeted therapies. Thus, the precise modulation of mitochondrial quality control and the correction of metabolic and oxidative imbalances may offer a promising approach for overcoming current therapeutic limitations. This review analyzes the current literature to summarize the pathological mechanisms and therapeutic strategies targeting mitochondrial dysfunction in HF, as a deeper understanding of these mechanisms may support the development of individualized treatment strategies for HF.

## 1. Introduction

Heart failure (HF) is the most prevalent form of fatal cardiovascular disease worldwide, with 55 million affected patients globally and steadily increasing [1] incidence and mortality rates in recent years, particularly among age thresholds for risk, etc. [2]. The pathogenesis of HF is complex and multifactorial, typically resulting from structural or functional abnormalities of the heart that give rise to characteristic clinical manifestations such as dyspnea, fatigue, and edema [3]. Given the heterogeneous and progressive nature of HF, therapeutic goals typically extend beyond symptom relief to delaying disease progression, improving patient prognosis, and enhancing quality of life. Current standard treatments include angiotensin-converting enzyme inhibitors, angiotensin receptor blockers (ARBs), β-adrenergic blockers, and cardiac resynchronization therapy [4,5,6]. Although these strategies can alleviate symptoms and prolong survival to some extent, their overall efficacy remains limited, particularly in elderly patients with multiple comorbidities. There thus remains an urgent need to further explore the pathogenesis of HF and to identify novel therapeutic targets for more effective disease management.

Disorders of myocardial energy metabolism and mitochondrial dysfunction are believed to constitute the core pathophysiological basis for the onset and progression of HF. L-carnitine and coenzyme Q10 (CoQ10) are essential for the maintenance of normal mitochondrial function, participate in adenosine triphosphate (ATP) synthesis via the mitochondrial electron transport chain, and exert potent antioxidant effects. L-carnitine mediates the transport of long-chain fatty acids into the mitochondria to complete β-oxidation, and its metabolic imbalance is closely associated with the progression of hypertensive HF. Loss of normal L-carnitine metabolic homeostasis can serve as an independent indicator of the severity of HF and has been proposed as a potential target candidate for targeted intervention [7].

CoQ10 can improve left ventricular ejection fraction and cardiac functional reserve in patients with HF and reduce the risk of cardiovascular death by optimizing cellular energy metabolism and alleviating oxidative stress injury. The randomized, double-blind Q-SYMBIO trial confirmed that CoQ10 supplementation effectively improves the prognosis of patients with chronic HF and reduces the incidence of adverse cardiovascular events [8]. A Cochrane systematic review summarizing multiple clinical studies has provided further high-quality evidence supporting the safety and efficacy of CoQ10 in the treatment of HF [9]. In addition, CarniQgel, a formulation combining L-carnitine and CoQ10, provides complementary support for mitochondrial metabolism. L-carnitine facilitates mitochondrial fatty acid uptake and oxidation, whereas CoQ10 supports electron transport and ATP production. Their combined antioxidant and cardioprotective effects may confer synergistic therapeutic benefits in HF. These effects further support the therapeutic potential of targeting key molecules involved in mitochondrial metabolism to modulate four major pathological processes underlying HF: impaired energy metabolism, oxidative stress, inflammation, and myocardial remodeling [10].

Mitochondria serve as the hub for cellular energy metabolism, playing indispensable roles in both catabolic and anabolic processes. In addition to ATP production, they regulate intracellular calcium (Ca^2+^) homeostasis and inflammatory responses and are critically involved in multiple cell death pathways [11]. As one of the most energy-demanding organs, the heart relies heavily on a continuous and efficient energy supply. Mitochondria occupy more than 30% of cardiomyocyte volume and generate approximately 95% of cellular ATP [12]. Cardiac energy demand dynamically adapts to workload, and excitation–contraction coupling is primarily driven by ATP generated via mitochondrial oxidative phosphorylation (OXPHOS) [13].

In HFrEF, impaired fatty acid β-oxidation, and mitochondrial dysfunction that compromises glucose oxidation, with glycolysis and ketone body oxidation only partially compensating for energy imbalance [14]. In HF with HFpEF, there is a mismatch between cardiac mechanical load and energy supply mediated by the mitochondrial tricarboxylic acid (TCA) cycle, particularly in patients with metabolic diseases in which mechanical load and metabolic overload collectively lead to metabolite accumulation, further impairing mitochondrial and cellular function [15]. Recent studies have revealed that PTEN-induced kinase 1 (PINK1) overexpression can protect against myocardial hypertrophy by negatively regulating the cyclic GMP–AMP synthase (cGAS)–stimulator of interferon genes (STING) signaling pathway [16]. Exercise intervention may ameliorate myocardial fibrosis, oxidative stress, and apoptosis by upregulating anti-inflammatory cytokines such as interleukin-10 (IL-10), thereby improving cardiac function [17]. Together, these observations highlight the critical importance of preserving mitochondrial function, regulating key kinase-mediated signaling pathways, and modulating inflammatory responses in alleviating HF progression across both reduced and preserved ejection fraction phenotypes.

The goal of this review is to overcome the limits associated with narrowly focused single-target studies by synthesizing recent research breakthroughs focused on mitochondrial dysfunction and exploring the synergistic regulatory mechanisms through which exercise and multi-molecule interventions can offer beneficial effects. Together, we aim to provide a summary of candidate biomarkers for early HF diagnosis and novel targets for clinical interventions, potentially facilitating the precision treatment of elderly patients with comorbidities.

## 2. Mechanistic Insights Into Mitochondrial Dysfunction in HF

**Fig. 1.**
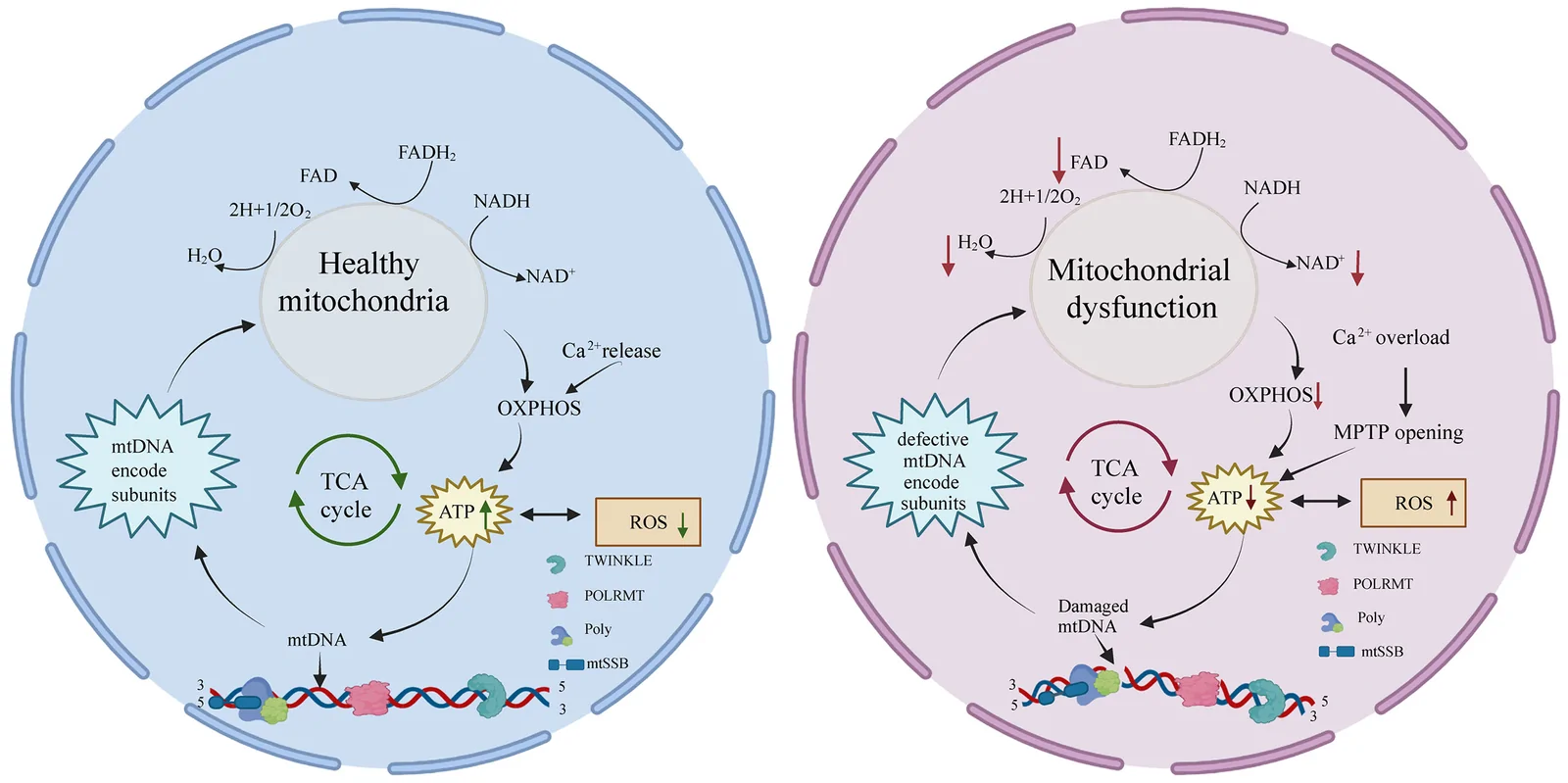
**Functional alterations of mitochondria in healthy and HF cardiomyocytes**. The left panel depicts healthy cells with normal mitochondrial function, featuring efficient OXPHOS, normal ATP production, and low ROS levels. The right panel shows HF cells with impaired mitochondrial function, suppressed OXPHOS, reduced ATP generation, and elevated ROS accumulation. HF, heart failure; OXPHOS, oxidative phosphorylation; ATP, adenosine triphosphate; ROS, reactive oxygen species; FAD, flavin adenine dinucleotide; FADH_2_, reduced flavin adenine dinucleotide; NADH, reduced nicotinamide adenine dinucleotide. The Figs. 1,2,3,4 were created with BioRender (https://www.biorender.com).

While they serve as the primary source of cardiomyocyte energy, mitochondria can, when impaired or otherwise dysfunctional, generate excessively high levels of reactive oxygen species (ROS), disrupt Ca^2+^ homeostasis, trigger ferroptosis, and initiate aberrant inflammatory responses, thereby accelerating myocardial injury and remodeling (Fig. 1). To systematically elucidate the pathological roles that mitochondria play in HF, this review offers an overview of key mechanisms, including metabolic remodeling, Ca^2+^ dysregulation, abnormal mitochondrial dynamics, mitochondrial DNA (mtDNA) damage, non-coding RNA (ncRNA)-mediated regulation, and ferroptotic induction, highlighting their interconnected contributions to HF progression to offer a theoretical foundation for subsequent mitochondria-targeted therapeutic strategies.

### 2.1 Metabolic Reprogramming

Fatty acids are first activated to fatty acyl-CoA and subsequently degraded through β-oxidation into acetyl-CoA, which enters the TCA cycle. This leads to the production of reduced flavin adenine dinucleotide (FADH_2_) and nicotinamide adenine dinucleotide (NADH), which donate electrons to the electron transport chain to generate ATP [18,19]. Glucose serves as a major energy substrate in the heart, with glycolysis-derived pyruvate entering mitochondria to fuel the TCA cycle and support OXPHOS–mediated ATP production [20]. Cardiomyocytes take up glucose through glucose transporter protein 1 (GLUT1) and GLUT4 transporters, which regulate glucose uptake. Notably, GLUT4 is modulated by both insulin signaling and myocardial contraction [21]. Enhanced glycolysis increases intracellular lactate and pyruvate levels, whereas their levels fall in the context of reduced glycolytic activity. During myocardial ischemia, impaired insulin signaling inhibits GLUT4 translocation, resulting in decreased glucose uptake and reduced glycolytic flux. Chronic metabolic remodeling of the myocardium ultimately impairs cardiac function and increases the risk of HF [22].

Disorders of myocardial glucose metabolism are frequently accompanied by dysregulated lipid metabolism [23]. The accumulation of excess lipid metabolites can trigger the activation of the IκB kinase (IKK)/nuclear factor kappa-B (NF-κB), c-Jun N-terminal Kinase (JNK)/activator protein-1 (AP-1), and protein kinase C (PKC) signaling pathways, promoting serine phosphorylation of insulin receptor substrate-1 (IRS-1) and thereby inhibiting protein kinase B (Akt) and phosphoinositide 3-kinase (PI3K) activity, ultimately reducing glucose uptake. In addition, cardiac uptake of non-esterified fatty acids (NEFAs) involves trans-endothelial transport and delivery to cardiomyocytes [24]. This process is mediated by the fatty acid transporter cluster of differentiation 36 (CD36), and CD36 deficiency consequently reduces lipid accumulation within cardiomyocyte mitochondria [25].

Through these distinct but interrelated processes, metabolic reprogramming serves as an upstream factor involved in the initiation of mitochondrial dysfunction. The resulting abnormalities in substrate supply and oxidative stress directly disrupt mitochondrial structure and function. Conversely, mitochondrial dysfunction further amplifies metabolic disturbances, creating a pathological feedback loop that lays the foundation for the onset and progression of HF.

### 2.2 Ca^2+^ Dysregulation

Ca^2+^ dyshomeostasis is a hallmark of HF pathogenesis. Ca^2+^ contributes to oxidative homeostasis by regulating antioxidant enzyme activity and ROS scavenging. Mitochondrial Ca^2+^ uptake primarily depends on the mitochondrial calcium uniporter (MCU) complex located in the inner mitochondrial membrane, which transports cytosolic Ca^2+^ into the mitochondrial matrix [26]. Hyperactivation of the MCU disrupts Ca^2+^ homeostasis. Ca^2+^ overload promotes ROS accumulation, opening of the mitochondrial permeability transition pore (mPTP), and oxidative stress–mediated injury [27]. These events lead to mitochondrial swelling, outer membrane rupture, and release of pro-apoptotic factors such as cytochrome c, further exacerbating ROS production and establishing a self-amplifying vicious cycle that drives cardiomyocyte dysfunction, apoptosis, and ultimately the progression of HF.

Under physiological conditions, Ca^2+^ both regulates excitation–contraction coupling and also participates in mitochondrial energy metabolism and cellular homeostasis [28]. Mitochondria are highly sensitive to ATP demands from the endoplasmic reticulum (ER). ATP levels decline upon inhibition of OXPHOS, whereas ER protein misfolding enhances mitochondrial ATP transfer to the ER, thereby disturbing cellular energy homeostasis [29]. Collectively, maintenance of mitochondrial Ca^2+^ homeostasis is closely linked to energy exchange between mitochondria and the ER. This synergistic effect is jointly regulated by the bidirectional interaction between calcium dysregulation and mitochondrial dysfunction. Imbalances in these two factors directly compromise the stability of the myocardial energy supply and contribute to the pathogenesis of HF. Calcium dysregulation and mitochondrial dysfunction establish a bidirectional regulatory relationship. They can act as upstream triggers that induce mitochondrial dysfunction while also serving as downstream effectors that exacerbate mitochondrial damage. This interplay couples these metabolic reprogramming-mediated pathological processes in a complex regulatory web, collectively driving the deterioration of myocardial function in HF.

### 2.3 Abnormal Mitochondrial Dynamics

Mitochondrial fusion and fission determine mitochondrial number, morphology, and activity. In energy-demanding cardiomyocytes, proper mitochondrial distribution and function rely on tightly coordinated dynamic processes and quality control mechanisms, as excessive fission can lead to deleterious mitochondrial fragmentation [30]. Dynamin-related protein 1 is a GTPase that controls mitochondrial fission by assembling on the mitochondrial membrane through interactions with receptors such as mitochondrial fission factor (Mff) and fission 1 (Fis1), thereby mediating mitochondrial constriction and division [31]. During fission, adaptor proteins recruit dynamin-related protein 1 (Drp1) to the outer mitochondrial membrane, particularly at sites of ER–mitochondria contact that mark pre-constriction regions. Drp1 oligomerizes and forms a GTP-dependent spiral structure around the outer mitochondrial membrane (OMM), constricting and severing mitochondria into two daughter organelles [32]. Cytosolic Drp1 translocation is mediated by its interaction with OMM adaptor proteins. Together, these components constitute the core fission machinery [33].

Optic Atrophy 1 (OPA1) is a key regulator of mitochondrial fusion. Although *OPA1* mutations are primarily associated with optic atrophy, emerging evidence indicates that OPA1 dysfunction can also disrupt mitochondrial energy metabolism and contribute to myocardial injury [34]. OPA1 promotes inner mitochondrial membrane fusion between adjacent mitochondria, thereby enhancing OXPHOS activity while also regulating cristae remodeling and overall mitochondrial function [35]. Peroxisome proliferator-activated receptor gamma coactivator-1β (PGC-1β) has been shown to regulate mitofusin 2 (Mfn2) at the transcriptional level through ERRα activation, thereby promoting mitochondrial fusion [36]. In PGC-1β knockout mice, reduced Mfn2 expression impairs mitochondrial fusion and leads to defects in myocardial energy metabolism, suggesting that Mfn2-mediated fusion deficiency may represent an important component of HF pathogenesis [36]. In addition to its role in mitochondrial fusion, Mfn2 also tethers mitochondria to the ER and maintains Ca^2+^ homeostasis. Loss of Mfn2 disrupts cardiomyocyte Ca^2+^ signaling and exacerbates myocardial injury [37]. Dysfunction or downregulation of fusion-related proteins such as OPA1 and Mfn2 further impairs mitochondrial network integrity, leading to dysregulated energy metabolism and Ca^2+^ homeostasis and thus constituting a critical driver of HF initiation and progression.

Mitophagy is one of the primary processes governing mitochondrial quality control by eliminating damaged mitochondria and thereby maintaining cardiac homeostasis [38]. Mitochondrial fission, fusion, and autophagy form a synergistic regulatory axis such that, during fission, damaged mitochondria are separated for subsequent autophagic degradation [39]. The PINK1-Parkin pathway is the primary pathway that controls Mitophagy in cardiomyocytes. When mitochondria are damaged, PINK1 accumulates and activates Parkin, triggering protein ubiquitination and autophagosome-mediated degradation [40]. In the context of HF, this pathway is frequently dysregulated.

Mitophagy acts as a double-edged sword in HF: adaptive mitophagy preserves mitochondrial homeostasis and function, whereas excessive mitophagy may lead to mitochondrial depletion and impaired cellular energy metabolism. The AMP-activated protein kinase-mammalian target of rapamycin (AMPK-mTOR) signaling pathway plays a critical regulatory role in this process, with AMPKα2 promoting mitochondrial autophagy to alleviate HF [41]. Mitochondrial dysfunction and abnormal calcium handling are closely linked to impaired mitochondrial quality control in HF. Ubiquitin-specific protease 30 (USP30), a deubiquitinating enzyme localized to the outer mitochondrial membrane, acts as a key negative regulator of mitophagy by counteracting Parkin-mediated ubiquitination signaling [42]. Impaired mitophagy is implicated in HFpEF progression, sparking interest in USP30 inhibition as a strategy to restore selective mitophagy. Aberrant USP30-mediated mitophagy dysregulation promotes mitochondrial injury and dysfunction in HFpEF, supporting USP30 as a promising therapeutic target.

### 2.4 mtDNA Damage-Mediated NLRP3 Inflammasome Activation

mtDNA is located within the mitochondrial matrix in close proximity to the respiratory chain, the primary source of ROS biogenesis. As it lacks any protective histones and possesses a relatively limited capacity for repair, mtDNA is highly susceptible to oxidative damage, and its mutation rate is significantly higher than that of nuclear DNA [43]. mtDNA encodes multiple essential subunits of the respiratory chain complexes that are indispensable for mitochondrial OXPHOS [44]. In patients with HF, mtDNA damage is markedly increased and is closely associated with oxidative stress and aberrant activation of inflammatory responses. Damaged mtDNA can activate innate immune signaling, thereby exacerbating myocardial injury. Excessive release of mtDNA into the cytosol initiates cyclic GMP-AMP synthase/stimulator of interferon genes (cGAS/STING) pathway signaling, leading to activation of the NOD-like receptor family pyrin domain containing 3 (NLRP3) inflammasome [45]. When active, the NLRP3 inflammasome promotes the maturation and secretion of pro-inflammatory cytokines IL-1β and IL-18, ultimately triggering chronic inflammatory responses [46]. Moreover, excessive acetylation of mitochondrial proteins facilitates NLRP3 inflammasome assembly, further aggravating inflammation. Nakahira et al. [47] demonstrated that, when stimulated with lipopolysaccharide (LPS) and ATP, wild-type macrophages exhibit enhanced mitochondrial ROS production, which promotes the cytosolic release and accumulation of mtDNA, thereby driving ROS-dependent NLRP3 inflammasome activation. In contrast, nuclear DNA remains unaffected [48]. In NLRP3-deficient cells, although mitochondrial ROS production is preserved, LPS- and ATP-induced mtDNA release is markedly attenuated, suggesting that the NLRP3 inflammasome plays a critical role in regulating mtDNA release and subsequent inflammatory responses [48]. Collectively, these findings highlight that oxidative mtDNA damage and its cytosolic release critically mediate NLRP3 inflammasome activation and chronic inflammation, forming a key pathological axis underlying HF progression.

### 2.5 Regulation by Non-Coding RNAs

**Fig. 2.**
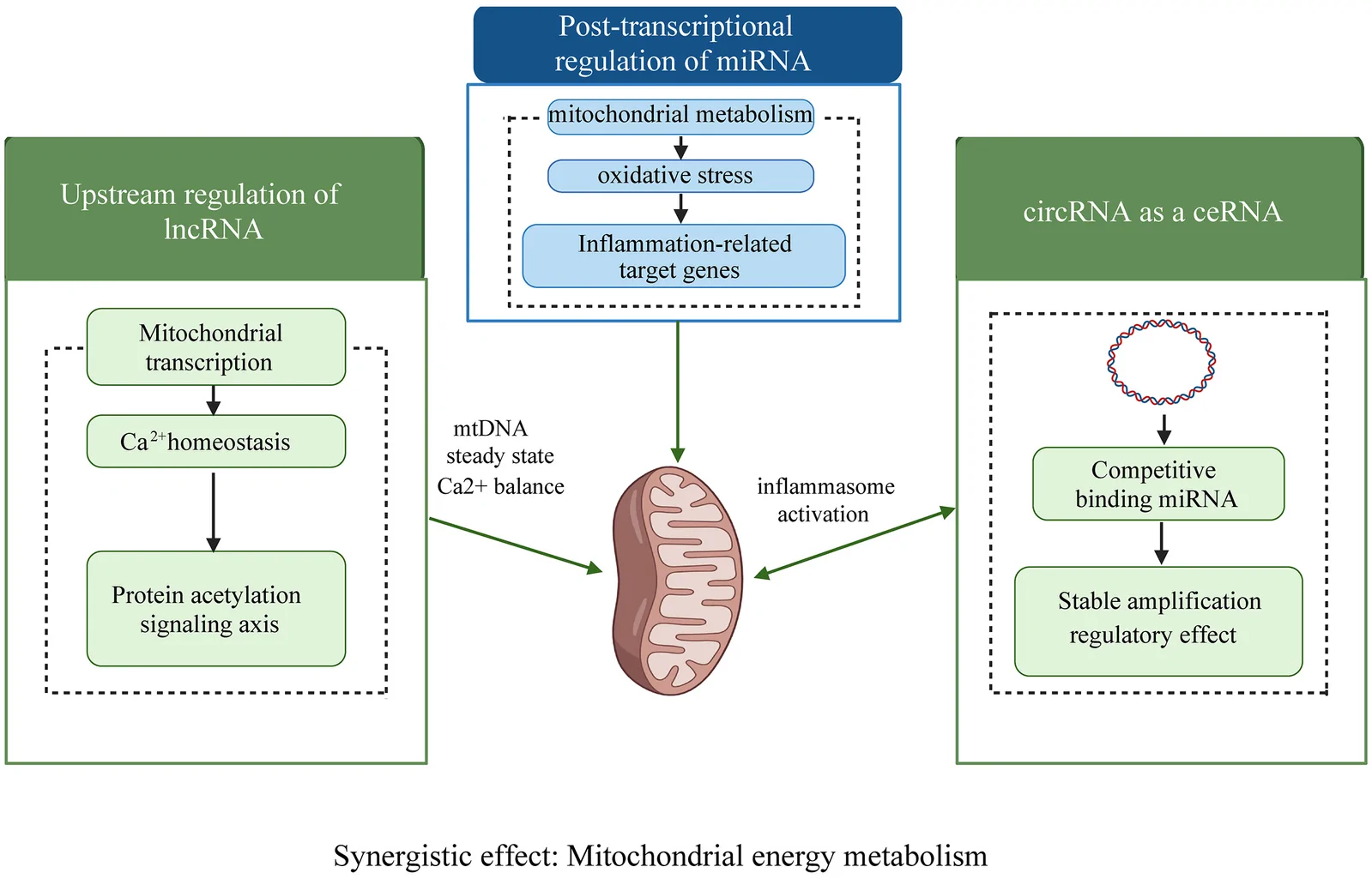
**Schematic diagram of the synergistic regulatory network of non-coding RNAs on mitochondrial pathways in HF**. The lncRNAs, miRNAs, and circRNAs form a hierarchical synergistic regulatory network. lncRNAs initiate upstream regulation, miRNAs perform post-transcriptional fine-tuning, and circRNAs exert stable regulation through ceRNA sponge effects. These three components collaboratively influence mitochondrial energy metabolism, Ca^2+^ homeostasis, ROS generation, and inflammatory activation, participating in the progression of mitochondrial dysfunction in HF.

Long non-coding RNAs (lncRNAs), microRNAs (miRNAs), and circular RNAs (circRNAs) are three major classes of ncRNAs that form an integrated and synergistic regulatory network that can shape HF development and progression. By precisely modulating mitochondrial function, energy metabolism, oxidative stress, and inflammatory responses, these ncRNAs participate in key aspects of HF pathogenesis, with their synergistic regulatory effects on mitochondrial pathways underscoring their potential relevance as targets for further studies focused on the diagnosis and treatment of HF (Fig. 2).

lncRNAs play essential roles in cardiac pathophysiology through their ability to regulate multiple aspects of mitochondrial metabolism. In cardiomyocytes, the lncRNA Cpat directly regulates the mitochondrial acetylation of citrate synthase, stabilizes the activity of key enzymes in mitochondrial metabolism, and preserves normal mitochondrial function and cardiac homeostasis [49]. Loss of Cpat function thus directly leads to the dysregulation of mitochondrial energy metabolism. Additionally, the lncRNA Mhrt has been shown to repress mitochondrial fission and attenuate mitochondrial ROS accumulation, thereby protecting against mitochondrial dysfunction and pathological cardiac remodeling [50]. During HF development, the abnormal expression and/or function of these lncRNAs can exacerbate extant imbalances in mitochondrial oxidative metabolism, insufficient energy production, and cardiomyocyte injury, driving the gradual progression from compensatory hypertrophy to decompensated HF [51]. Targeting mitochondrial metabolism via lncRNA-associated regulatory networks may offer novel mechanistic insights and therapeutic strategies for HF. A recent study shows that Glucose Metabolism Regulatory Small Protein (GMRSP), a small peptide encoded by lncRNA H19, attenuates aortic remodeling by regulating cellular metabolic reprogramming [52], indicating that lncRNAs and their encoded products can modulate cardiac disease progression at the metabolic level.

miRNAs possess significant potential as relevant biomarkers for HF. As post-transcriptional regulatory molecules, they precisely shape mitochondrial metabolism, Ca^2+ ^homeostasis, and inflammatory responses by targeting mRNAs that encode mitochondrion-associated genes [53]. For example, miR-195 regulates myocardial energy metabolism by modulating SIRT3 expression and mitochondrial protein acetylation. miR-21 is highly induced in cardiac macrophages under HF conditions [54,55], promoting macrophage polarization toward a pro-inflammatory phenotype and enhancing cardiac fibroblast activation, which exacerbates myocardial inflammation and fibrosis. Moreover, overexpression of miR-25 in the failing heart downregulates SERCA2a expression, disrupts intracellular calcium homeostasis, and ultimately compromises cardiomyocyte contractile function and overall cardiac performance [56].

circRNAs are RNA molecules with a covalently closed-loop structure generated through the back-splicing of exons or introns [57]. Recent evidence suggests that circRNAs, including circSMAD4, circPostn, and circPAN3, play pivotal regulatory roles in cardiac fibrosis and myocardial infarction [58]. Further evidence has shown that circRNAs in the epicardial adipose tissue of HF patients may contribute to HF progression by modulating mechanisms associated with metabolic dysregulation, thereby providing additional potential therapeutic targets [59].

lncRNAs, miRNAs, and circRNAs form a synergistic regulatory pattern in which lncRNAs serve as core upstream regulators, miRNAs act as intermediate fine regulators targeting mitochondrial-related proteins to precisely modulate mitochondrial metabolism, Ca^2+^ homeostasis, and inflammatory responses, and circRNAs function as a stable regulatory layer, enhancing the stability of the overall regulatory network while controlling mitochondrial apoptosis and metabolic remodeling. Specifically, in the context of HF, the lncRNA MALAT1 acts as a molecular sponge to sequester miR-125b, a miRNA that directly targets the mitochondrial fusion protein Mfn2; this sequestration relieves miR-125b-mediated repression of Mfn2, thereby promoting mitochondrial fusion, improving mitochondrial function, and attenuating cardiomyocyte injury [60,61]. Additionally, MALAT1 overexpression inhibits the PI3K/Akt signaling pathway, while restoring miR-150-5p levels with miR-150-5p mimics reduces cell apoptosis, increases autophagy, and reactivates this pathway, supporting that aerobic exercise improves CHF cardiac function by inhibiting MALAT1, potentially via the miR-150-5p/PI3K/Akt pathway [62]. Meanwhile, circ-Cdr1as exerts potent cardioprotective effects by functioning as a molecular sponge for miR-7, thereby upregulating the expression of its downstream target KLF4. This regulatory axis promotes the anti-inflammatory phenotypic transformation of macrophages, reduces myocardial inflammatory injury, alleviates mitochondrial dysfunction and structural damage in cardiomyocytes, and ultimately attenuates infarct size and preserves left ventricular function [63]. As a ceRNA, circHIPK3 functions as a molecular sponge for miR-93, a well-established regulator implicated in diverse pathological processes. miR-93 has been shown to inhibit cardiomyocyte hypertrophy by targeting SIRT4. In combination with its interaction with circHIPK3, these findings suggest the existence of a coordinated regulatory axis involving lncRNA MALAT1, miR-93, and circHIPK3 [64,65].

These interrelated functions directly control the onset and progression of mitochondrial dysfunction in HF through the joint regulation of critical processes, highlighting the pivotal role of ncRNAs in HF pathogenesis. Further efforts to target these transcripts in a therapeutic context are thus warranted.

### 2.6 Ferroptosis

Ferroptosis is a regulated, non-apoptotic form of cell death characterized by intracellular iron overload and excessive lipid peroxidation [66]. This process is closely associated with distinct mitochondrial structural and functional alterations, including mitochondrial shrinkage, outer membrane rupture, loss of cristae, and increased membrane density [67]. Accumulating evidence indicates that ferroptosis is a key driver of various cardiac pathologies, including myocardial ischemia/reperfusion injury, cardiomyopathy, and HF.

Ferroptosis is typically accompanied by depletion of glutathione and inhibition of glutathione peroxidase 4 (GPX4) activity, leading to systemic pro-inflammatory responses, coronary microvascular dysfunction, myocardial structural damage, and the eventual impairment of cardiac function [68]. Within the complex regulatory network governing cardiomyocyte ferroptosis, multiple enzymatic systems act synergistically to promote its initiation and progression. Among them, phosphorylase kinase G2 enhances the availability of iron for lipoxygenases, catalyzing the bis-allylic oxidation of polyunsaturated fatty acids and generating lipid peroxides. If these lipid peroxidation products are not efficiently eliminated, they trigger and amplify a lipid peroxidation chain reaction, thereby compromising membrane integrity and promoting ferroptotic cell death [69,70]. In addition to this phosphorylase kinase catalytic subunit gamma 2 (PHKG2) axis, cytochrome P450 enzymes, which utilize heme as a cofactor, initiate phospholipid peroxidation in cardiomyocytes and contribute to ferroptotic initiation [71]. When antioxidant defenses collapse due to dysfunction of these enzymatic oxidative pathways, intracellular lipid peroxidation is continuously amplified, leading to cardiomyocyte death characterized by ferroptosis.

Intracellular iron homeostasis and redox metabolism are tightly regulated by both enzymatic and non-enzymatic mechanisms. Expansion of the labile iron pool markedly increases cellular susceptibility to ferroptosis. Excess Fe^2+^ generates large amounts of ROS through the Fenton reaction, directly initiating oxidative stress–mediated damage [72]. The glutathione (GSH)/GPX4 axis represents a central pathway by which cardiomyocytes resist ferroptosis [73,74]. There is strong evidence that ferroptosis plays a critical role in the onset and progression of HF resulting from myocardial infarction and cardiomyopathy, marking it as a promising therapeutic target for HF [75].

**Fig. 3.**
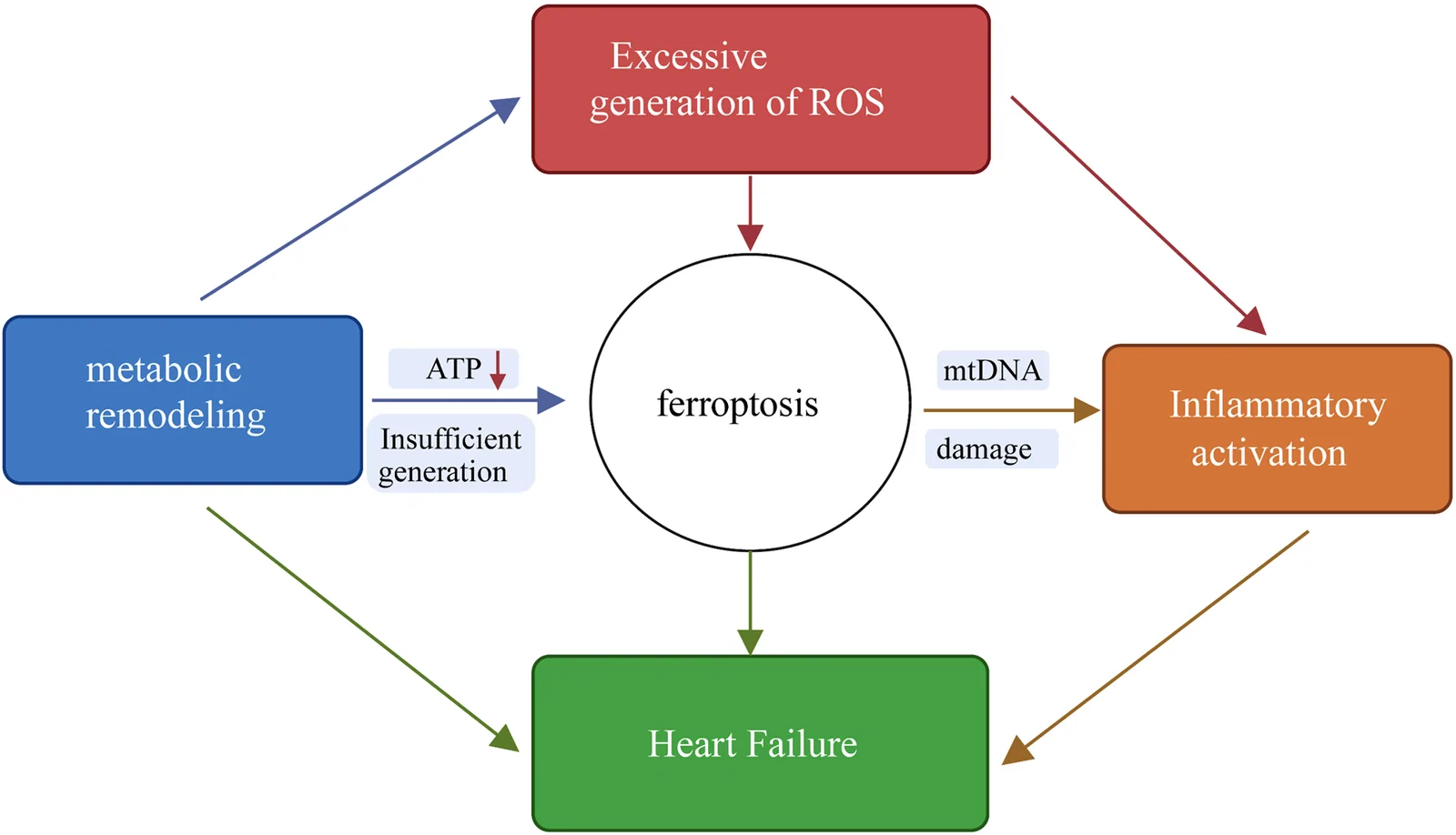
**Ferroptosis serves as the central hub**. Metabolic remodeling contributes to excessive ROS generation and insufficient ATP production, thereby promoting ferroptosis. Excessive ROS generation further promotes ferroptosis, mtDNA damage and inflammatory activation. These interconnected processes collectively drive the progression of HF.

Despite this strong theoretical foundation for targeting ferroptosis in HF, many challenges continue to hamper the translation of these findings into a clinical context. The greatest of these challenges is the lack of specificity of ferroptosis inhibitors. Current established inhibitors (e.g., Ferrostatin-1, Liproxstatin-1) predominantly act on common targets in the ferroptosis pathway by disrupting lipid peroxidation and chelating iron ions, such that they lack any specificity for cardiac tissues or cardiomyocytes [76,77]. When administered systemically, these drugs may thus exert nonspecific effects on healthy metabolically active tissues such as the liver and kidneys, interfering with their iron metabolism and cellular survival and thus impairing normal physiological functions. Off-target risks further limit the clinical application of known ferroptosis inhibitors. The ferroptosis pathway is characterized by extensive cross-communication with other cell death pathways, including both apoptosis and pyroptosis, hampering the ability of most inhibitors to solely impact one form of cell death. Their use may therefore lead to unintended modulation of other cell death pathways while inhibiting cardiomyocyte ferroptosis, resulting in adverse effects such as abnormal myocardial tissue repair and inflammatory dysregulation [78]. Moreover, certain inhibitors may disrupt systemic iron metabolism by affecting key enzymes related to iron homeostasis (e.g., transferrin, ferritin), potentially causing iron overload or iron deficiency that can further exacerbate cardiac injury or induce other systemic diseases [79]. At present, ferroptosis inhibitors targeting cardiac diseases remain confined to the preclinical research space. Enhancing tissue specificity of inhibitors, reducing off-target risks, establishing scientific dosing regimens, and developing efficacy evaluation systems will be crucial for the meaningful clinical translation of ferroptosis-related targets (Fig. 3).

## 3. Mitochondria-Targeted Therapeutic Strategies

Mitochondria-targeted therapies have shown broad therapeutic potential in the management of HF. In recent years, several pharmacological agents have demonstrated cardioprotective effects by improving mitochondrial function and attenuating oxidative stress and inflammatory responses.

### 3.1 Mitochondrial Targeting Activity of Established Drugs

In addition to their metabolic benefits, sodium–glucose cotransporter 2 (SGLT2) inhibitors have emerged as important modulators of inflammatory pathways in several disease contexts. SGLT2 inhibitors, such as dapagliflozin, canagliflozin, and empagliflozin, significantly reduce both the expression levels and activity of NLRP3 inflammasome components and pro-inflammatory cytokines, including IL-1β, IL-6, and tumor necrosis factor-alpha (TNF-α), in diabetic animal models [80].

Specific drugs have been further shown to alleviate oxidative stress and apoptosis in cardiomyocytes by restoring mitochondrial membrane potential and inhibiting mPTP opening, while also suppressing left ventricular collagen deposition and reversing cardiac fibrotic remodeling [81]. The mineralocorticoid receptor antagonist (MRA) spironolactone, for instance, mitigates abnormal aldosterone activation, improves left ventricular diastolic dysfunction in HF patients, and slows the progression of cardiac fibrosis and inflammation [82,83]. As a calcium sensitizer with ATP-sensitive potassium channel opening activity, it exhibits strong mitoprotective effects. It facilitates the stabilization of the mitochondrial ultrastructure via two distinct mechanisms: activation of myocardial mitochondrial ATP-sensitive potassium channels, as well as a connexin 43‑mediated signaling pathway. This process has been demonstrated to effectively inhibit ferroptosis in an obese hypertensive HF model, achieving sustained enhancement of myocardial contractility and mitigating ischemic injury [84].

Similarly, statins, selective β-adrenergic blockers, and SGLT2 inhibitors exert indirect anti-ferroptotic effects by attenuating lipid peroxidation and limiting iron overload in stressed cardiomyocytes. Collectively, these findings indicate that targeting ferroptosis represents a common mechanism underlying the cardioprotective effects of various pharmacotherapies, supporting its use as a unified strategy for the treatment of different types of HF [85,86].

### 3.2 Multi-Target Mitochondrial Regulation by Traditional Chinese Medicine

Traditional Chinese medicine has also demonstrated cardioprotective effects in cardiovascular diseases, with accumulating evidence highlighting its multifaceted regulatory effects on mitochondrial homeostasis and cellular stress responses [87]. Beyond general antioxidative and anti-inflammatory actions, a growing body of mechanistic research, particularly from network pharmacology and experimental validation, indicates that several classic Traditional Chinese Medicine (TCM) herbs exert direct and targeted effects on mitochondrial quality control systems, including mitochondrial fusion–fission dynamics, mitophagy, respiratory chain function, and redox balance [88,89].

For example, integrative network pharmacology analyses have revealed that Astragalus membranaceus, Salvia miltiorrhiza, Poria cocos, and Aconitum carmichaelii modulate key inflammatory signaling pathways by suppressing the expression and release of critical inflammatory mediators such as IL‑6, TNF, Vascular Endothelial Growth Factor A (VEGFA), Prostaglandin-Endoperoxide Synthase 2 (PTGS2), and NOS2. Importantly, these herbal interventions also act at the transcriptional level to restore the dynamic equilibrium of mitochondrial fusion and fission, which is frequently dysregulated in failing cardiomyocytes. By fine‑tuning the expression of core regulators governing mitochondrial morphology, these agents help prevent excessive mitochondrial fragmentation and structural collapse, thereby preserving mitochondrial integrity [79,90].

Furthermore, these TCM components have been shown to enhance the activity of mitochondrial respiratory chain complexes, improve OXPHOS efficiency, and reduce the overproduction of mtROS. Collectively, these effects alleviate pathological cardiac remodeling, attenuate maladaptive inflammatory cascades, and re‑establish cardiac energy metabolic homeostasis in the stressed myocardium. Given their ability to simultaneously target inflammation, oxidative stress, and mitochondrial dysfunction—core pathological drivers of HF—these TCM‑based strategies exhibit distinctive translational value as potential adjunctive therapies for both HFrEF and HFpEF.

### 3.3 Non-Pharmacological Interventions

Regular endurance or resistance training can significantly enhance mitochondrial biogenesis and increase mtDNA copy number by activating the PGC-1α pathway, thereby improving myocardial energy reserve capacity under conditions of stress [91,92]. Additionally, exercise enhances the efficiency of mitochondrial autophagy, clears damaged mitochondria, and maintains intracellular environmental stability [93].

Specific nutritional interventions (such as supplementation with Omega-3 polyunsaturated fatty acids, CoQ10, and short-chain fatty acids) can directly replenish mitochondrial substrate supply and strengthen the antioxidant defense system [94]. Dietary interventions regulating the gut microbiota-mitochondrial axis not only improve the metabolic microenvironment but also synergistically enhance the efficacy of pharmacotherapy, providing a scientific basis for developing nutritional pharmacology-based combination regimens [95].

## 4. Limitations

Although remarkable progress has been made in the study of HF and mitochondrial dysfunction, with core mechanisms including mitochondrial structural remodeling, impaired energy metabolism, oxidative stress injury, and dysregulated mitophagy having been extensively characterized, several limitations and unresolved key issues remain in this field that warrant further validation and biochemical characterization [96].

First, existing HF models fail to fully recapitulate the true pathological microenvironment present in humans. Current basic research relies heavily on conventional *in vitro* cell models and standardized animal models. While valuable for mechanistic investigation, these models cannot replicate the complex etiology, multiple comorbidities, and clinical heterogeneity of human HF. Consequently, the efficacy of mitochondrial-targeted interventions is often unsatisfactory when translated to the clinic, leading to a substantial gap between basic research and clinical application [97]. Second, the complexity and crosstalk of the mitochondrial regulatory network have not been fully elucidated. Mitochondrial dysfunction is not an isolated event, but rather forms an intricate regulatory network involving multiple pathways, including calcium homeostasis, autophagy, protein synthesis, lipid metabolism, and gene expression [98]. Most studies published to date have opted to focus on a single molecule or pathway, thus lacking a systematic understanding of the overall regulatory network. In addition, there remains a lack of detailed characterization of mitochondrial signaling crosstalk between cardiomyocytes and non-cardiomyocytes, such as fibroblasts, endothelial cells, and immune cells, as well as the distinct forms of mitochondrial damage evident in different forms of HF (e.g., ischemic, dilated, hypertrophic, etc.) [99]. Finally, individual heterogeneity has been insufficiently studied in this context, and research on precision therapy remains scarce. HF is defined by marked individual differences in terms of genetic background, etiology, and comorbidities, with similarly high degrees of heterogeneity in terms of mitochondrial functional phenotypes and mechanisms of injury. Most extant studies rely on population-averaged analyses without precise stratification guided by multi-omics or radiomics approaches, making it difficult to develop individualized mitochondrial-targeted therapeutic strategies. The most effective means of integrating molecular mechanisms with clinical phenotypes and facilitating large-scale clinical validation remains a critical bottleneck for the widespread clinical application of mitochondrial-targeted therapies in HF.

## 5. Conclusions

**Fig. 4.**
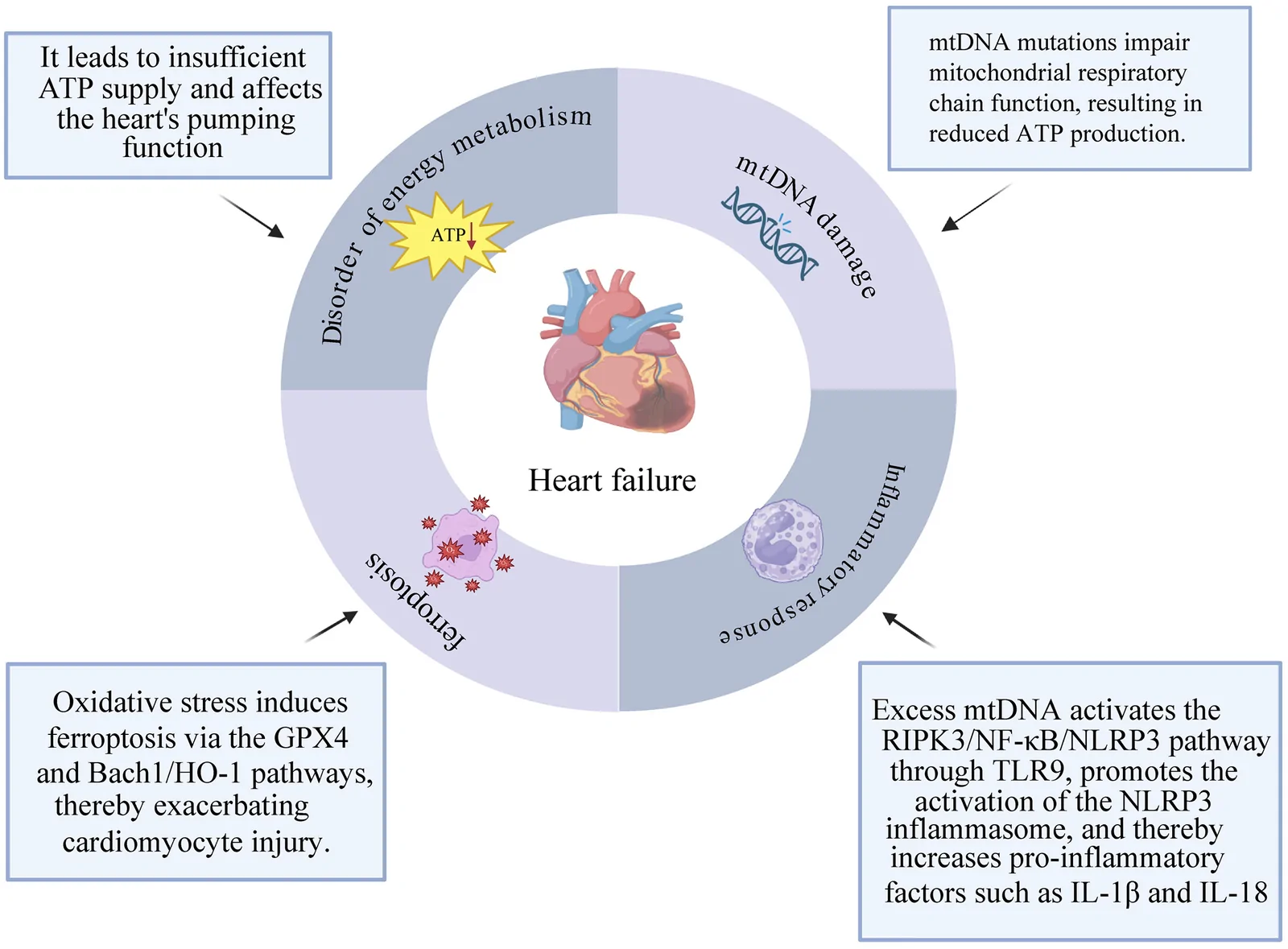
**Key roles and interactions of metabolic dysregulation, ferroptosis, mtDNA damage, and inflammatory signaling in the pathogenesis and progression of HF**.

This review offers a comprehensive overview of current knowledge regarding the role of mitochondria in maintaining cardiac energy metabolism under both physiological and pathological conditions, with a particular emphasis on the central role of mitochondrial dysfunction in the initiation and progression of HF and its therapeutic implications. Key pathways involved in HF pathogenesis, including mtDNA damage, ferroptosis, mitochondrial dynamic imbalance, and inflammatory signaling, have been discussed at length (Fig. 4). Specifically, during HF progression, transcriptional alterations in mitochondrial energy metabolism enzymes are accompanied by redox imbalance and metabolic signaling dysregulation, leading to a metabolic shift from fatty acid oxidation toward glycolysis, insufficient ATP production, and excessive ROS accumulation. Elevated ROS further induces mtDNA mutations, activates TLR9/NF-κB and NLRP3 inflammasome pathways to promote chronic inflammation, and triggers aberrant mPTP opening, which exacerbates oxidative stress and forms a vicious feedback cycle, while cardiac immune cell infiltration (particularly macrophages) mediates cardiomyocyte apoptosis, hypertrophic remodeling, fibroblast proliferation, and extracellular matrix remodeling to perpetuate HF progression. In addition, the regulatory effects of mitochondrial communication, metabolic alterations, and external stressors on mitochondrial structure and function in HF have been highlighted. Given the complexity of HF pathophysiology, single-target interventions are unlikely to fully reverse disease progression; thus, future therapeutic strategies should focus on multi-targeted approaches (including metabolic reprogramming, antioxidant therapy, regulation of mitochondrial dynamics, and modulation of inflammatory signaling) to restore mitochondrial homeostasis through multi-dimensional strategies. Further elucidation of the dynamic structural and functional changes of cardiomyocyte mitochondria and the crosstalk among signaling pathways in HF will facilitate the development of more precise and effective therapeutic interventions.
